# A New Journal

**Published:** 1873-04

**Authors:** 


### Editorial.

### *A New Journal.*

We welcome to our exchange list THE SANITARIAN: a Monthly Journal. A. N. BELL, M.D., Editor. Published by A. S. Barnes & Company, New York and Chicago. Pp. 48. Thirty cents single copy—$3 a year in advance. The object in chief of the SANITARIAN will be to awaken public attention to the extent of the field of sanitary science and indicate how beneficently it may be cultivated. The Prospectus remarks:

"This will be done by showing the amount of ill health and mortality from preventable causes of disease; by pointing out the nature of those causes, and the way in which they operate; by showing that such causes are removable; and by exhibiting improved health, longevity and happiness as the fruits of their removal.

"The laws of physiology and general pathology will be kept in view, as the basis of health; and by which hygiene constitutes a department of science which the medical profession can advantageously share with the public, or apply to individuals according to circumstances. The detail of these relations will involve questions of manifold significance, and many of them of the utmost importance to human health.

"The practical questions of State Medicine: the health of armies and navies, marine hygiene, quarantine, civic cleanliness, water supply, drainage and sewerage. Sanitary architecture: light, space, warming and ventilation. Climate and domicile: epidemic, endemic and hereditary diseases. Occupation: exercise and habits, food and beverages, in all varieties of quality and quantity. In short, whatever thing, condition or circumstance is in rapport with, or antagonistic to, the most perfect culture of mind and body will be considered legitimate matter for the SANITARIAN to discuss, advocate, condemn or reject at the *bar of health*."

Among the indorsers of the Editor, and his object, we find such well known names as Drs. Stephen Smith, S. O. Vanderpoel (Health Officer, Port of New York), Willard Parker, S. G. Armor, Wm. A. Hammond, J. Ordronaux, A. Flint, Jr., etc., etc.

It is unnecessary for us to remark that the most of the existing journals ostensibly devoted to hygiene and sanitary movements, are in fact travesties. Their main idea seems to be to tickle the public fancy, with crude mixtures of stale truths, and dogmas none the less offensive because "sent into this breathing world scarce half made up" rather than worm-eaten or fossilized. There are two classes in the public which such journals as the one before us may well enlighten—the one which trusts in everything, and beyond all due bounds, in medical men, and the other which is largely or altogether sceptical as to their value. There is vastly more in medical science (including sanitary science) than many believe, and there is vastly less than either professional or lay optimists have a notion.

